# The Diagnosis of Gastric Ulcer

**Published:** 1901-02-09

**Authors:** 


### THE DIAGNOSIS OF GASTRIC ULCER.

MR. MAYO ROBSON,[1] speaking on the subject of the diagnosis of gastric ulcer from cancer of the stomach, says that if HCl is present, especially if in excess, the fact is decidedly in favour of the ailment being ulcer and not cancer. On the other hand, the absence of free HCl from the vomit, although not affording positive evidence of cancer, certainly favours that diagnosis. Again, while in ulcer vomiting gives marked relief to pain, this is not always the case in cancer, and in regard to the smell of the vomited matter he says that "a yeasty smell is characteristic of dilated stomach, a habitual fœtid odour of cancer, and a fæculent odour of intestinal obstruction." Profuse and clotted blood is usually the result of ulcer, and blood small in quantity and of coffee-ground appearance of cancer; but this does not always hold good—indeed, sometimes the opposite occurs. To ascertain the size of the stomach a small teaspoonful of tartaric acid dissolved in a half-tumblerful of warm water may be swallowed, followed by a teaspoonful of sodium bicarbonate dissolved in another half-tumblerful of water. The resulting evolution of carbonic acid gas distends the stomach and enables one to ascertain the presence of a dilated stomach, the size of it, the existence of hour-glass contraction, the position of the upper and lower borders, and the approximate position and thickness of the pylorus. Although the presence of a tumour is very suggestive of cancer it must be remembered that in chronic ulcer of the stomach a very considerable tumour may form and simulate cancer. Such a tumour, however, is usually smoother than the nodular tumour of cancer, and is markedly tender. The duration of the disease is also a symptom which sometimes affords help, cancer being a disease of months, while chronic ulcer with tumour may last for years. Sometimes an exact diagnosis can only be arrived at by an exploratory incision, this being especially the case in the earlier stages of cancer, and in those cases of chronic ulcer with tumour in which it is a question whether or not cancer has supervened on ulceration. Before making such an incision, however, one must feel clear that the operation can be performed without adding seriously to the risk to life, and that it is possible that some good will result from it.

[1] Brit. Med. Jour., Feb. 2.

